# Preparation and Functional Assessment of Composite Chitosan-Nano-Hydroxyapatite Scaffolds for Bone Regeneration

**DOI:** 10.3390/jfb3010114

**Published:** 2012-02-13

**Authors:** Benjamin T. Reves, Jessica A. Jennings, Joel D. Bumgardner, Warren O. Haggard

**Affiliations:** Biomedical Engineering Department, University of Memphis, Memphis, TN 38152, USA; E-Mails: jjnnings@memphis.edu (J.A.J.); jbmgrdnr@memphis.edu (J.D.B.); whaggrd1@memphis.edu (W.O.H.)

**Keywords:** bone regeneration, chitosan, hydroxyapatite, tissue engineering

## Abstract

Composite chitosan-nano-hydroxyapatite microspheres and scaffolds prepared using a co-precipitation method have shown potential for use in bone regeneration. The goal of this research was to improve the functional properties of the composite scaffolds by modifying the fabrication parameters. The effects of degree of deacetylation (DDA), drying method, hydroxyapatite content and an acid wash on scaffold properties were investigated. Freeze-dried 61% DDA scaffolds degraded faster (3.5 ± 0.5% mass loss) than air-dried 61% DDA scaffolds and 80% DDA scaffolds, but had a lower compressive modulus of 0.12 ± 0.01 MPa. Air-dried 80% DDA scaffolds displayed the highest compressive modulus (3.79 ± 0.51 MPa) and these scaffolds were chosen as the best candidate for use in bone regeneration. Increasing the amount of hydroxyapatite in the air-dried 80% DDA scaffolds did not further increase the compressive modulus of the scaffolds. An acid wash procedure at pH 6.1 was found to increase the degradation of air-dried 80% DDA scaffolds from 1.3 ± 0.1% to 4.4 ± 0.4%. All of the formulations tested supported the proliferation of SAOS-2 cells.

## 1. Introduction

Approximately five to ten percent of bone fractures will result in delayed healing or non-union [1]. In Europe, insufficient bone healing results in socioeconomic losses of around 14.7 billion euros each year [2]. Although autografts and allografts are commonly used to treat these troublesome fractures, a number of drawbacks with these procedures have generated interest in the development of bone graft substitutes [3,4,5,6]. These bone graft substitutes are designed to provide a favorable matrix to which osteoblasts can attach, proliferate and subsequently produce new bone. These materials are also expected to provide some mechanical support and stability to the fracture site until osteogenesis occurs. In addition, these bone scaffolds should degrade in a timely manner so that new bone can completely fill the defect site [7,8,9,10]. Many of these scaffolds also serve as drug delivery vehicles for the local release of growth factors to further augment fracture healing [2,11,12]. 

Our laboratory group has previously developed chitosan-nano-hydroxyapatite scaffolds for use in bone regeneration [13,14,15]. The scaffolds are prepared by fusing composite microspheres together to form porous scaffolds. Chitosan is a carbohydrate co-polymer containing glucosamine and *N*-acetyl-D-glucosamine monomers [16,17]. Chitosan displays a number of properties including biocompatibility, degradability, mucoadhesiveness and an ability to promote wound healing that have led to the development of chitosan sponges, films, gels, beads, etc. for use in various biomedical applications [16,17,18,19,20]. Hydroxyapatite is the main inorganic component of bone and has been used to improve osseoeintegration of implants and in bone graft substitutes [20,21,22,23]. We have previously demonstrated the potential of these composite chitosan-nano-hydroxyapatite scaffolds to serve as bone graft substitutes both *in vitro* and *in vivo* [13,14,15]. Although the scaffolds are highly biocompatible and we have observed new bone in direct contact with the scaffolds *in vivo*, more extensive new bone formation appears to be prevented due to slow degradation of the scaffolds [13,14]. We believe that the osteogenic capacity of the scaffolds can be further enhanced by also improving their degradation profile. 

An important parameter of chitosan is degree of deacetylation (DDA), which is defined as the ratio of deacetylated glucosamine units to the total number of monomers [16,17]. DDA has an effect on a number of chitosan properties, including crystallinity, degradation and mechanical strength. Since the lower number of acetyl residues allow for tighter packing of the polymer chains, high DDA chitosan will be more crystalline than lower DDA chitosan (if all other parameters are equal) [19,24,25]. High DDA chitosan materials are more rigid and stronger than low DDA chitosan materials [26,27], but degrade more slowly [24,27,28,29,30]. In previous studies, composite scaffolds were prepared using 92.3% DDA chitosan [13,14,15]. This high DDA was chosen due to its good mechanical properties. Using lower DDA chitosan to fabricate composite scaffolds may result in increased degradation.

Our laboratory group has also demonstrated that increased surface area can be obtained by freeze-drying (lyophilization) [15]. Increased surface area may promote degradation by exposing more of the surface of the scaffolds to lysozyme and by increasing fluid uptake. Chitosan is soluble in weak organic acids due to protonation of the amine group on the glucosamine residues and the pK_a_ is approximately 6.5 [31]. Washing composite microspheres in a mildly acidic solution before fusing them into scaffolds was identified as another potential method to increase scaffold degradation. Lower DDA chitosan, lyophilization and a mild acid wash were evaluated for increased degradation of composite scaffolds.

Although the main goal of this research is to improve the degradation profile of the scaffolds to allow more extensive bone ingrowth, the other characteristics required of bone scaffolds should still be met. The scaffolds need sufficient mechanical properties to provide space maintenance at the fracture site and to prevent collapse of the scaffold pores [8]. Maintaining porosity is vital so that cells can migrate to the interior of the scaffold and also to enable proper nutrient/waste exchange throughout the scaffold [7,32,33]. The scaffolds must provide a favorable surface for osteoblast attachment and proliferation [8]. Chitosan-nano-hydroxyapatite composite scaffolds had previously been shown to have both superior mechanical properties and induce more favorable cellular responses compared to plain chitosan scaffolds [13,14]. The addition of more hydroxyapatite to the composite scaffolds could possibly improve the properties of the scaffolds even more. In this research, fabrication parameters including chitosan DDA, microsphere drying method, hydroxyapatite content and the use of a mild acid wash were investigated to fabricate composite chitosan-nano-hydroxyapatite scaffolds with improved properties for bone regeneration.

## 2. Experimental Section

### 2.1. Phase I: Effects of DDA and Drying Method

**Figure 1:**
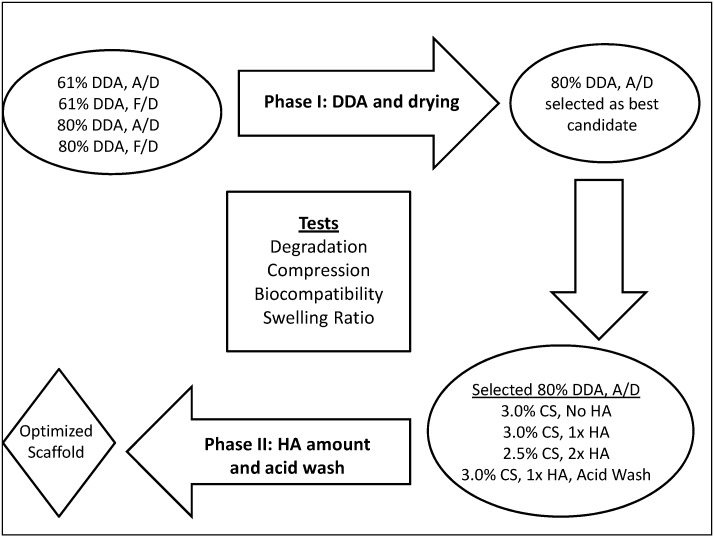
Experimental design used for scaffold characterization. Degradation of air-dried and freeze-dried 61% and 80% Degree of Deacetylation (DDA) microspheres was also determined. All groups underwent SEM analysis. A/D: air-dried; F/D: freeze-dried; CS: chitosan; HA: hydroxyapatite.

In Phase I of this research, the effects of DDA and drying method were evaluated. Air-dried (A/D) and freeze-dried (F/D) 61% and 80% DDA scaffolds were prepared (Figure 1). 

#### 2.1.1. Microsphere Fabrication

Composite chitosan-nano-hydroxyapatite microspheres were prepared using a co-precipitation method as previously described [13,15]. Briefly, solutions containing 61% DDA (MW = 220 kDa) or 80% DDA (MW = 260 kDa) chitosan (Primex, Siglufjörður, Iceland), 0.1M CaCl_2_ and 0.06M NaH_2_PO_4_ (referred to as 1× HA, Ca:P ratio = 1.67) were prepared in 2 volume percent (vol. %) acetic acid (Table 1). 

**Table 1:** Chitosan solutions used to make composite beads. Note: Dissolved in 200 mL of 2 vol.% acetic acid. CS: Chitosan, HA: Hydroxyapatite.

Microsphere Type	Chitosan (g)	CaCl_2_●2H_2_0 (g)	NaH_2_PO_4_●H_2_0 (g)
**61% DDA**: 3.5% CS, 1× HA	7.00	2.94	1.66
**80% DDA**: 3.0% CS, 1× HA	6.00	2.94	1.66

A precipitation solution (pH = 13) containing 20 weight percent (wt.%) NaOH, 30 wt.% methanol and 50 wt.% water was prepared. Using a syringe pump, the chitosan solution was added dropwise through 16G needles into the precipitation solution and microspheres immediately formed. The microspheres were stirred in the precipitation solution for 24 hours to allow crystalline hydroxyapatite to form. The microspheres were then washed in deionized (DI) water until a neutral pH (<7.5) was achieved.

Microspheres were either air-dried (A/D) or freeze-dried (F/D). Air-drying was performed by placing neutralized beads in plastic weigh boats and drying them in a chemical fume hood at room temperature. Freeze-drying was performed by placing neutralized (still hydrated) beads in plastic weigh boats and pre-freezing at −20 °C in a laboratory freezer for two hours. The beads were then freeze-dried in a 2.5 L Labconco lyophilizer for 48 hours.

#### 2.1.2. Scaffold Fabrication

Porous scaffolds were prepared by fusing dried microspheres together. The microspheres were rinsed in 1wt.% acetic acid for approximately ten seconds in a ceramic sieve. Excess acid was removed using a vacuum. This very brief acid wash gently dissolves the outer layer of the beads and makes them adherent. Using a laboratory spatula, the microspheres were then placed in 12 mm diameter polystyrene tubes to form cylindrical scaffolds. Only very slight pressure was applied to the microspheres with the spatula as they were placed in the polystyrene tubes. After approximately one minute, the scaffolds were removed from the molds and allowed to air-dry. Following rehydration, the scaffolds can be cut into any height as desired.

### 2.2. Phase II: Effects of Hydroxyapatite Content and 2-(N-morphilino)ethanesulfonic (MES) Acid Wash

#### 2.2.1. Microsphere and Scaffold Fabrication

Based on the data obtained in Phase I, air-dried 80% DDA scaffolds were determined to be good candidates for bone regeneration. In Phase II, the following groups of air-dried 80% DDA beads/scaffolds were prepared to determine the effect of hydroxyapatite content and a mild acid wash: 3.0% CS, No HA; 3.0%, 1× HA; 2.5% CS, 2× HA; and 3.0% CS, 1× HA, acid wash. As seen in Table 2, No HA refers to solutions containing only chitosan. 2× HA denotes solutions with 0.2 M CaCl_2_ and 0.12 M NaH_2_PO_4_ (Ca:P ratio = 1.67).

**Table 2:** Chitosan solutions used to make air-dried 80% DDA beads. Note: Dissolved in 200 mL of 2 vol. % acetic acid. CS: Chitosan, HA: Hydroxyapatite. *Also prepared using 2-(N-morphilino)ethanesulfonic (MES) acid wash.

Microsphere Type	Chitosan (g)	CaCl_2_●2H_2_0 (g)	NaH_2_PO_4_●H_2_0 (g)
3.0% CS, No HA	6.00	0.00	0.00
3.0% CS, 1× HA*	6.00	2.94	1.66
2.5% CS, 2× HA	5.00	5.88	3.31

In Phase II, all of the microspheres were air-dried. Scaffolds were prepared by the method previously described in Section 2.1.2.

#### 2.2.2. MES Acid Wash

Some of the 3.0% CS, 1x HA beads underwent a mild acid wash before being air-dried (Table 2). A 40 mM 2-(N-morphilino)ethanesulfonic acid (MES) solution was prepared and the pH was raised to 6.1 using concentrated NaOH. Neutralized microspheres (80% DDA, 3.0% CS, 1× HA) were added to the MES solution. The microspheres were allowed to wash for ten minutes and the pH was maintained at 6.1 by adding additional MES powder. The microspheres were removed, washed in DI water, washed in 70% and 95% ethanol and placed in a chemical fume hood to dry. After completely drying, the microspheres were then washed in 1× phosphate buffered saline for thirty minutes and allowed to completely air-dry again. Scaffolds were prepared by the method previously described in Section 2.1.2.

### 2.3. Characterization

#### 2.3.1. Scanning Electron Microscopy

Scanning electron micrograph (SEM) images of composite microspheres and scaffolds were obtained using a Philips XL30 environmental microscope. Samples were coated with 30 nm of Au/Pd before imaging to make them conductive. Microsphere size and surface topography were evaluated using SEM images.

#### 2.3.2. Microsphere and Scaffold Degradation

Immediately prior to starting the degradation study, microsphere samples were placed in a convection oven at 50 °C to mitigate the effects of ambient humidity. After one hour, the microspheres were removed from the convection oven and weighed. An amount of 4mL of degradation solution containing 100 μg/mL lysozyme (MP Biomedicals, Cat. No. 100834) + antibiotics/antimycotic (1 unit/mL penicillin, 1 μg/mL streptomycin and 0.25 μg/mL amphotercin B) in DI water was added to each sample. Lysoyzme is the main enzyme responsible for chitosan degradation *in vivo* [29,34]. The samples were placed in an incubator at 37 °C and the degradation solution was refreshed every three days. After one month, the microspheres were allowed to air-dry and were then heated in a convection oven at 50 °C for one hour. The microspheres were weighed and the percent weight change was determined using the following equation:

(Initial weight − final weight)/(Initial weight) × 100(1)

Scaffold degradation was performed in the same manner as microsphere degradation. Scaffolds were completely submerged in 6 mL of 100 μg/mL lysozyme + antibiotics/antimycotic. After one month, the percent weight change was measured.

#### 2.3.3. Compression Testing

The compressive moduli of scaffolds were determined using an Instron load frame (Model # 33R 4465). Since the scaffolds will become hydrated after implantation, they were rehydrated in DI water. 61% DDA scaffolds were rehydrated for four hours. The interior beads of 80% DDA scaffolds were not completely hydrated after four hours, so these scaffolds were rehydrated for eight hours. The diameter of the scaffolds varied depending on the microsphere type and the rehydrated scaffolds were sectioned so that the height:diameter ratio was maintained at approximately 1.5. The scaffolds were then compressed at a strain rate of 0.1 min^−1^ until 50% strain was achieved. The compressive modulus was determined using the initial linear portion of the stress-strain curve.

#### 2.3.4. Swelling Ratio

The swelling ratio of scaffolds was determined. Pre-weighed scaffolds were placed in 10 mL of deionized water and put in an incubator at 37 °C. After 24 hours, the scaffolds were removed and pat-dried to remove any excess moisture on the surface. The scaffolds were re-weighed and the swelling ratio was determined using the following equation:

(Final weight − Initial weight)/(Initial weight) × 100(2)

#### 2.3.5. Biocompatibility

The biocompatibility of each microsphere type was evaluated using osteoblast-like SAOS-2 cells. Cells in McCoy’s 5A media supplemented with 15% fetal bovine serum and antibiotics/antimycotic were seeded at a density of 2.2 × 10^5^ cells/sample onto microspheres in Transwell inserts in 24-well plates. The cells were incubated at 37 °C with 5% CO_2_. After allowing cells to attach for three hours, the inserts were transferred to empty wells and cell attachment was evaluated using the CellTiter-Glo assay (Promega). A standard curve relating luminescence output from the assay to cell number was constructed by seeding cells at a known concentration. The remaining samples were returned to the incubator after media refreshment. Cell numbers were determined on Day 2 and Day 5.

### 2.4. Statistical Analysis

Mean ± standard deviations are presented. One-factor ANOVA with Bonferroni post-test was performed to determine statistical differences, with p < 0.05 considered significant. Two-factor ANOVA with Bonferroni post-test was used to analyze the biocompatibility data.

## 3. Results

### 3.1. SEM Images

**Figure 2:**
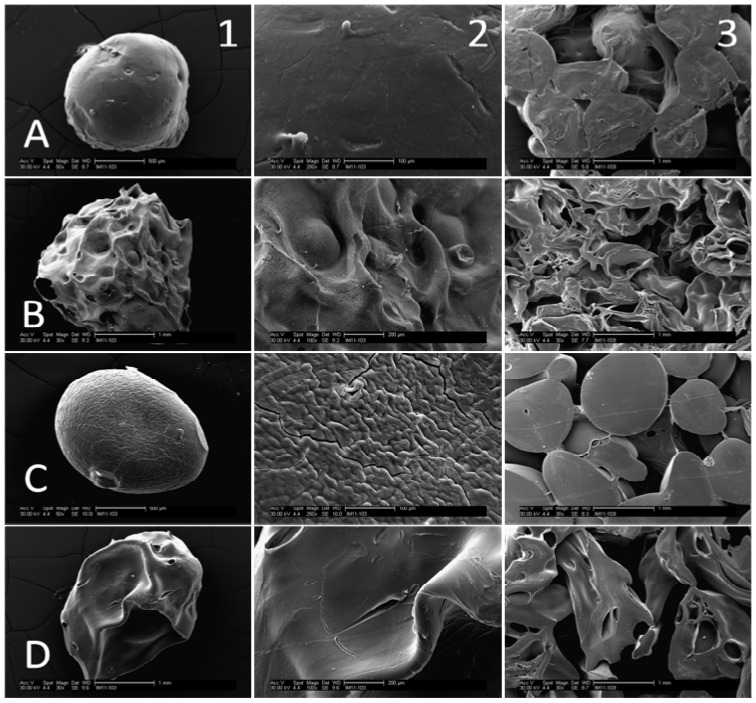
SEM micrographs of air-dried (A/D) and freeze-dried (F/D) 61% and 80% DDA microspheres and scaffolds. **A**: 61% DDA, 3.5% CS, 1x HA, A/D; **B**: 61% DDA, 3.5% CS, 1× HA, F/D; **C**: 80% DDA, 3.0% CS, 1× HA, A/D; **D**: 80% DDA, 3.0% CS, 1× HA, F/D. **1**: Microsphere at low magnification-50× (A,C) and 30× (B,D); **2**: Microsphere at high magnification-250× (A,C) and 100× (B,D); **3**: Scaffolds at 30× magnification.

**Figure 3:**
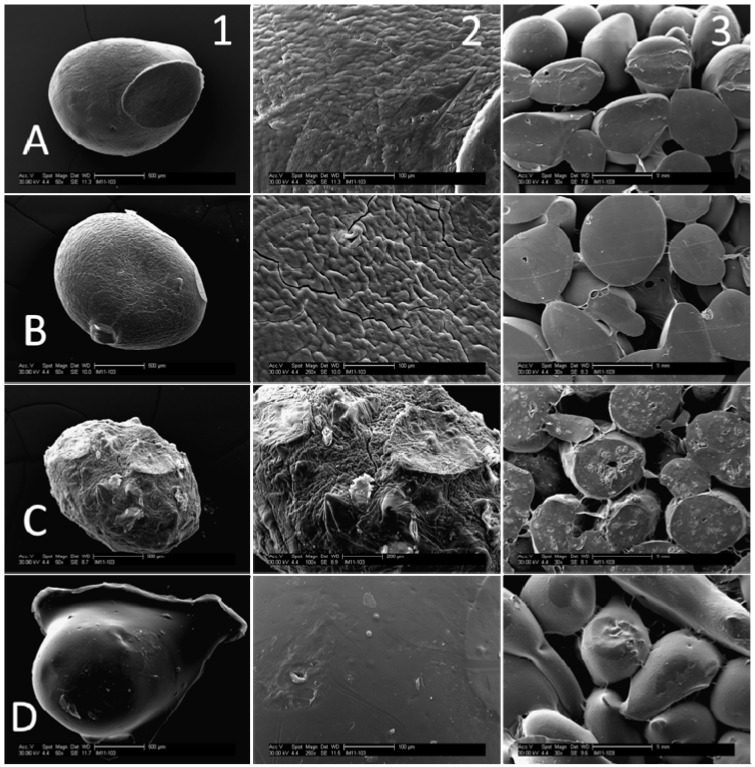
SEM micrographs of air-dried 80% DDA microspheres and scaffolds. **A**: 3.0% CS, No HA; **B**: 3.0% CS, 1× HA; **C**: 2.5% CS, 2× HA; **D**: 3.0% CS, 1× HA, MES acid wash. **1**: Microsphere at low magnification—50×; **2**: Microsphere at high magnification—250× (A,B,D) and 100× (C); **3**: Scaffolds at 30× magnification.

Processing parameters were found to have an effect on microsphere properties (Figure 2 and Figure 3). Air-dried microspheres are smaller and spherical compared to freeze-dried microspheres which are larger and somewhat teardrop-shaped. Air-dried 61% DDA beads have a very smooth surface. By contrast, some cracks and surface roughness are visible on air-dried 80% beads. Larger scale surface features are visible on both 61% and 80% DDA freeze-dried microspheres. When the amount of hydroxyapatite was increased, the surface of the air-dried 80% DDA beads became considerably rougher. The MES acid wash altered the shape of the beads. The edges of the acid-washed beads appeared rounded due to slight dissolution; interestingly, the surface of these beads was considerably smoother than that of 80% DDA beads which had not undergone the acid wash. For all bead types, porous scaffolds were successfully prepared by fusing microspheres together. 

### 3.2. Microsphere Degradation

**Figure 4:**
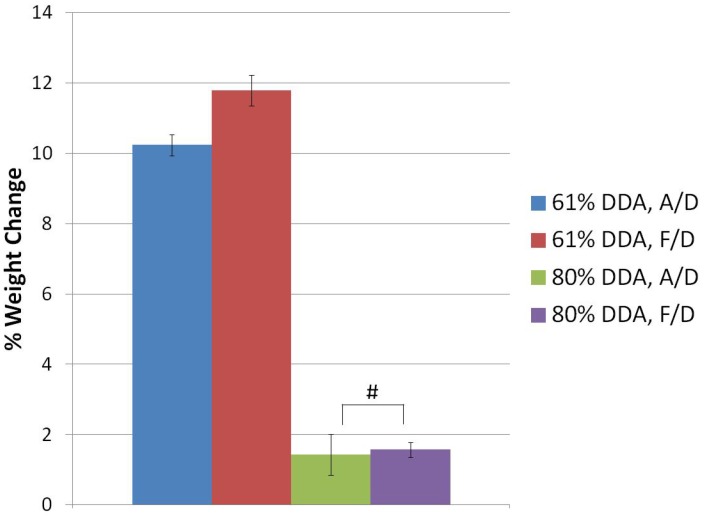
Degradation of 61% DDA and 80% DDA microspheres. 61% DDA microspheres are 3.5% CS, 1× HA. 80% DDA microspheres are 3.0% CS, 1× HA. Statistical differences existed between all groups except 80% DDA air-dried and 80% DDA freeze-dried beads; # represents no statistical difference; N = 3.

As seen in Figure 4, 61% DDA microspheres were found to degrade approximately five times faster than 80% DDA microspheres. Freeze-drying minimally increased the degradation of 61% DDA beads (p < 0.001) but did not increase the degradation of 80% DDA beads (p = 0.70).

### 3.3. Phase I: Effects of DDA and Drying Method

Overall, the degradation rate of scaffolds was much lower than that of microspheres (Table 3). Freeze-dried 61% DDA scaffolds had the highest degradation with 3.5 ± 0.5% weight change after one month, which was statistically different from all of the other groups (p < 0.001). The degradation rates of the other three scaffold groups were statistically similar.

**Table 3:** Degradation, compression testing and swelling ratio of composite scaffolds.

Scaffold Type	Degradation (% wt. change) (N = 4)	Compressive Modulus (MPa) (N = 3)	Swelling Ratio (%) (N = 4)
**61% DDA, A/D**	1.4 ± 0.5	0.67 ± 0.06	148.3 ± 11.7 ^b^
**61% DDA, F/D**	3.5 ± 0.5 ^a^	0.12 ± 0.01	267.1 ± 15.3 ^b^
**80% DDA, A/D**	1.3 ± 0.1	3.79 ± 0.51 ^a^	88.5 ± 1.9 ^b^
**80% DDA, F/D**	0.8 ± 0.3	0.81 ± 0.14	116.5 ± 6.9 ^b^

^a^: Statistically different from all other groups; ^b^: Statistically different from all other groups.

Scaffolds composed of air-dried 80% DDA microspheres had the largest compressive modulus (Table 3), which was statistically different from all of the other groups (p < 0.001). None of the scaffolds fractured during compression testing. The 61% DDA scaffolds had higher swelling ratios than the 80% DDA scaffolds and freeze-drying increased the swelling ratio (Table 2). Statistical differences in swelling ratio existed between all of the groups.

**Figure 5:**
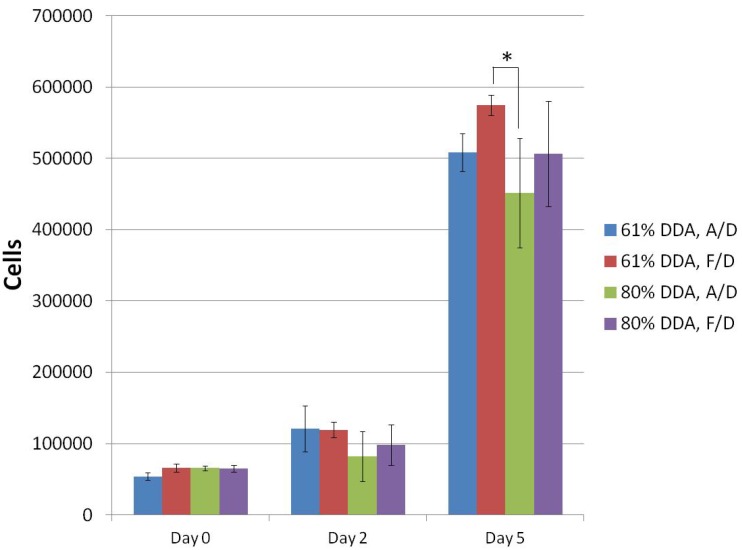
Biocompatibility of composite microspheres using SAOS-2 cells. *represents statistical significance; N = 4.

Both air-dried and freeze-dried 61% and 80% DDA microspheres were found to be biocompatible (Figure 5). Cell numbers increased at each timepoint. The only statistically significant difference between the bead types was between 61% DDA freeze-dried beads and 80% DDA air-dried beads on Day 5 (p < 0.001). 

### 3.4. Phase II: Effects of Hydroxyapatite Content and MES Acid Wash

Since all of the scaffold groups displayed similar biocompatibility and degradation characteristics in Phase I, compression testing was used to select a candidate for additional studies. Due to their considerably higher compressive modulus of 3.79 ± 0.51 MPa (Table 3), air-dried 80% DDA scaffolds with 3.0% CS, 1× HA were selected as the most promising formulation for further enhancement. In Phase II, the effects of hydroxyapatite content and an MES wash on air-dried 80% DDA scaffolds were determined.

**Figure 6:**
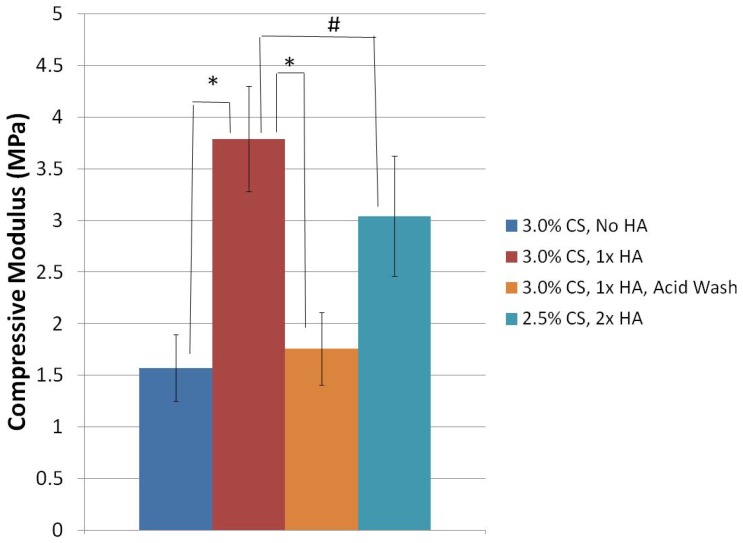
Compressive moduli of air-dried 80% DDA scaffolds. Scaffolds were compressed at a strain rate of 0.1 min-1 until 50% strain was achieved. None of the scaffolds fractured during testing. The compressive moduli were calculated from the initial linear portion of the stress-strain curve. *represents statistical significance. # represents no statistical difference. N = 3.

Scaffolds composed of 3.0% CS, 1× HA were found to still have the highest compressive modulus of all groups tested (Figure 6). However, this value was not significantly different from scaffolds prepared with 2.5% CS, 2× HA, which had a value of 3.04 ± 0.58 MPa (p = 0.535). A decrease in the compressive modulus to 1.57 ± 0.32 MPa was observed in the scaffolds prepared without hydroxyapatite (p < 0.001). The compressive moduli of 3.0% CS, 1× HA scaffolds which had undergone the mild acid wash was significantly reduced to 1.76 ± 0.35 MPa (p < 0.001). 

Scaffolds undergoing the acid wash were found to have a degradation of 4.4 ± 0.4% after one month (Table 4), which was significantly higher than all of the other groups (p < 0.001). The acid wash increased the swelling ratio of the scaffolds. Increasing the hydroxyapatite content was found to decrease the swelling ratio.

Scaffolds undergoing the acid wash were found to have a degradation of 4.4 ± 0.4% after one month (Table 4), which was significantly higher than all of the other groups (p < 0.001). The acid wash increased the swelling ratio of the scaffolds. Increasing the hydroxyapatite content was found to decrease the swelling ratio.

**Table 4:** Degradation and swelling ratio of 80% DDA scaffolds.

Scaffold Type	Degradation (% wt. change) (N = 4)	Swelling Ratio (%)
		(N = 4)
**3.0% CS, No HA**	−0.3 ± 0.4 ^a^	159.1 ± 1.9 ^b^
**3.0% CS, 1× HA**	1.3 ± 0.1	88.5 ± 1.9 ^b^
**2.5% CS, 2× HA**	1.7 ± 0.2	66.1 ± 0.9 ^b^
**3.0% CS, 1× HA, Acid Wash**	4.4 ± 0.4 ^a^	180.4 ± 3.3 ^b^

^a^: statistically different from all other groups; ^b^: statistically different from all other groups.

**Figure 7:**
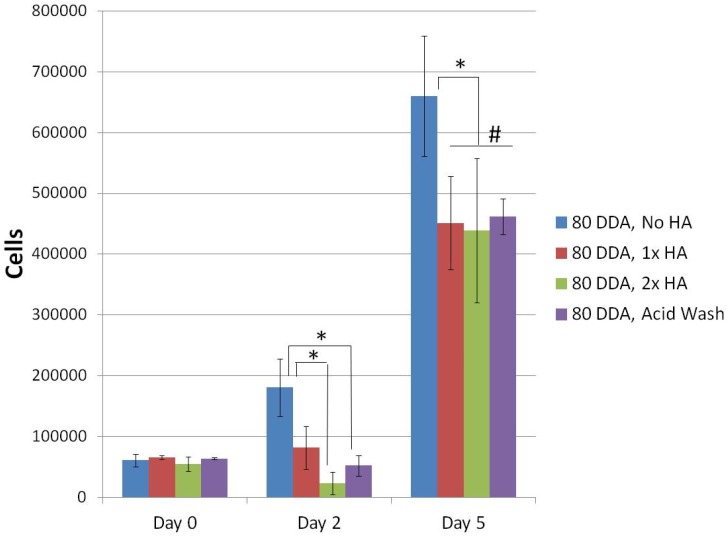
Biocompatibility of 80% DDA microsphere formulations using SAOS-2 cells. * represents statistical significance; # represents no statistical difference; N = 4.

All of the 80% DDA microsphere variations were found to be biocompatible (Figure 7). No differences in attachment were found on Day 0. On Day 2, all of the groups had statistically similar numbers of cells compared to Day 0, except for the No HA group which had significantly more cells (p = 0.009). The No HA group had significantly more cells than the 2× HA group (p = 0.001) and acid wash group (p = 0.010) on Day 2. By Day 5, cell numbers had increased for all bead types compared to previous timepoints. The No HA group had the most cells compared to the other groups and the other three groups were not statistically different.

## 4. Discussion

Composite chitosan-nano-hydroxyapatite microspheres were successfully prepared using a co-precipitation method. SEM analysis revealed that fabrication parameters had an effect on bead characteristics including size, shape and surface roughness. In addition, porous composite scaffolds composed of fused microspheres were successfully fabricated. The degradation, mechanical, biocompatibility and swelling ratio properties of the modified microspheres and scaffolds were evaluated to determine the best formulation for bone regeneration.

### 4.1. Phase I: Effects of DDA and Drying Method

Interestingly, the concentration of chitosan that can be used to fabricate beads successfully is within a narrow range. When the chitosan solution is dripped into the precipitating solution, the chitosan solution cannot be too thick or too thin. If the chitosan solution is too thick, long strands or very teardrop-shaped beads instead of spherical microparticles will precipitate. In contrast, if the chitosan solution is too thin, small fragments of chitosan will precipitate instead of microspheres. In these studies, 61% DDA beads could only be prepared using a 3.5% chitosan solution due to the reasons discussed above. When 80% DDA beads were prepared, a 3.5% chitosan solution produced very elongated particles. However, spherical 80% DDA beads could be fabricated when 3.0% and 2.5% chitosan solutions were used.

The increased degradation of 61% DDA microspheres compared to 80% DDA microspheres was expected. Chitosan films, beads and sponges have been shown to degrade faster when prepared with lower DDA [24,27,28,29,30]. Lim *et al.* observed the weight half-lives of 52.6%, 56.1% and 62.4% DDA chitosan beads to be 9.8, 27.3 and 56 days compared to weight half-lives of over 84 days for 71.7, 81.7 and 93.5% DDA beads [29]. The fabrication method for preparation of these beads was not disclosed. There is some debate as to why high DDA chitosan degrades more slowly. Some researchers have suggested that the crystalline nature of high DDA chitosan prevents lysozyme from easily accessing the glycosidic bonds between the polymer chains, resulting in slower degradation [19,25]. Others researchers have suggested that the binding site of lysozyme require a certain number of acetylated residues to be present for lysozyme to be able to degrade chitosan [28,30]. 

Since freeze-drying was previously shown to increase the surface area of microspheres by more than 200× [15] and freeze-drying increased the swelling ratio, it is somewhat surprising that freeze-drying only increased the degradation of 61% DDA beads by less than two percent. Furthermore, scaffolds were observed to degrade more slowly than microspheres. The freeze-dried 61% DDA scaffolds degraded the fastest but only exhibited 3.5 ± 0.5% weight change over one month. Presumably, these differences in degradation between beads and scaffolds are a surface area issue. 

Mechanical properties are an important consideration for bone regeneration constructs, with air-dried 80% DDA scaffolds being found to have the highest compressive modulus. This is most likely due to their higher crystallinity and lower swelling ratio. It should be noted that even though the 61% DDA scaffolds were rehydrated for only four hours, they had lower compressive moduli than the 80% DDA scaffolds which had been rehydrated for eight hours. Other researchers have also observed increased compressive strength and modulus when using higher DDAs [26,27]. The 3.79 ± 0.51 MPa compressive moduli of the air-dried 80% DDA scaffolds is considerably higher than the moduli of approximately 10 kPa or less reported for many chitosan scaffold preparations [10,35,36]. Furthermore, air-dried 80% DDA scaffolds exhibited the best handling properties. Handling refers to the ease with which we believe a surgeon can grab, manipulate and place the scaffold into a bone defect. For these reasons, we felt that air-dried 80% DDA scaffolds held the most promise as bone tissue scaffolds of the formulations tested the next phase of the investigation focused on further improving the air-dried 80% DDA scaffolds.

### 4.2. Phase II*:* Effects of Hydroxyapatite Content and MES Acid Wash

Other researchers have also observed that the addition of nano-hydroxyapatite decreases the swelling ratio [10,37] and increases the compressive moduli and strength of chitosan scaffolds [10,38]. However, addition of too much hydroxyapatite may have negative effects. In the current study, the compressive modulus of air-dried 80% DDA scaffolds with 3.0% CS, 1× HA (3.79 ± 0.51 MPa) was higher than that of both 3.0% CS, No HA (1.57 ± 0.32 MPa) and 3.0% CS, 2× HA (2.51 ± 0.16 MPa, (data not shown) scaffolds. Similar to our results, Zhang *et al.* observed that increasing the hydroxyapatite content of chitosan-nano-hydroxyapatite scaffolds improved compressive strength to a point, but further increases in hydroxyapatite content decreased the compressive strength [38]. In addition to altering structure at a molecular level, too much hydroxyapatite may prevent the beads from being able to be fused together as soundly, resulting in poorer mechanical properties. In fact, beads containing 4× HA could not even be fused together into scaffolds (data not shown). Somewhat surprisingly, the compressive modulus of scaffolds made with 2.5% CS, 2× HA beads was not significantly different from that of 3.0% CS, 1× HA. For this reason, air-dried 80% DDA scaffolds were prepared with 2.5% CS, 2× HA instead of 3.0% CS, 2× HA during Phase II of the experiments.

In this study, plain 80% DDA microspheres without hydroxyapatite promoted increased proliferation of SAOS-2 cells when compared with composite microspheres with hydroxyapatite. This result was unexpected, since we previously observed greater cell proliferation on composite scaffolds compared to plain scaffolds [13,14]. These previous investigations were performed using human fetal osteoblast cells and human embryonic palatal mesenchymal stem cells. The SAOS-2 cells used in the current study may respond differently to hydroxyapatite than the other cell types. 

The MES acid wash was found to increase degradation of air-dried 80% DDA beads to 4.4 ± 0.4%. During the acid wash, the beads appeared to shrink slightly and felt slightly “sticky”-suggesting mild dissolution. The degradation rate of acid-washed beads could potentially be increased even further by using a lower pH or increasing the wash time. When compared to freeze-dried 61% DDA scaffolds, the 80% DDA acid-washed scaffolds had both higher degradation and compressive modulus. 

Depending on the intended application, the properties of the chitosan-nano-hydroxyapatite microspheres can be altered by changing the fabrication parameters as demonstrated in this research. For application in bone tissue engineering, using a combination of bead types to prepare scaffolds may be a promising approach. A combination of fast-degrading 61% DDA freeze-dried beads or acid-washed beads could be used in conjunction with slower-degrading but mechanically stronger 80% air-dried beads. The fast-degrading beads could deliver a growth factor such as BMP-2 and then degrade, increasing the available space for tissue ingrowth. The slower-degrading beads would provide mechanical stability at the fracture site until new bone had developed and then be resorbed more slowly. The ratio of beads types could be altered to give an optimized scaffold for an intended musculoskeletal application. 

## 5. Conclusions

Microspheres with 61% DDA exhibited more than 10% degradation after one month compared with less than 2% for 80% DDA microspheres. Freeze-drying minimally increased the degradation of 61% DDA microspheres. Scaffold degradation was found to be lower than microsphere degradation. The 3.8 ± 0.5 MPa compressive modulus of air-dried 80% DDA scaffolds made them good potential candidates for bone regeneration. Increases in hydroxyapatite content did not increase the compressive modulus of the 80% air-dried scaffolds. A brief acid wash in an MES solution increased the degradation of air-dried 80% DDA scaffolds. All of the composite microspheres tested demonstrated good biocompatibility with SAOS-2 osteoblast-like cells. This study demonstrated the ability to modify the functional properties of composite chitosan-nano-hydroxyapatite scaffolds by altering the DDA, drying method, hydroxyapatite content and using an MES wash.
